# An Efficient CNN-Based Deep Learning Model to Detect Malware Attacks (CNN-DMA) in 5G-IoT Healthcare Applications

**DOI:** 10.3390/s21196346

**Published:** 2021-09-23

**Authors:** Ankita Anand, Shalli Rani, Divya Anand, Hani Moaiteq Aljahdali, Dermot Kerr

**Affiliations:** 1 Chitkara University Institute of Engineering and Technology, Chitkara University, Rajpura 140401, India; aankitaanand2719@gmail.com; 2 Department of Computer Science and Engineering, Lovely Professional University, Phagwara 144411, India; divyaanand.y@gmail.com; 3 Faculty of Computing and Information Technology, King Abdulaziz University, Jeddah 37848, Saudi Arabia; Hmaljahdali@kau.edu.sa; 4 Intelligent Systems Research Centre, University of Ulster, Londonderry BT48 7JL, UK

**Keywords:** healthcare, 5G-IoT, deep learning, malware, CNN, malimg

## Abstract

The role of 5G-IoT has become indispensable in smart applications and it plays a crucial part in e-health applications. E-health applications require intelligent schemes and architectures to overcome the security threats against the sensitive data of patients. The information in e-healthcare applications is stored in the cloud which is vulnerable to security attacks. However, with deep learning techniques, these attacks can be detected, which needs hybrid models. In this article, a new deep learning model (CNN-DMA) is proposed to detect malware attacks based on a classifier—Convolution Neural Network (CNN). The model uses three layers, i.e., Dense, Dropout, and Flatten. Batch sizes of 64, 20 epoch, and 25 classes are used to train the network. An input image of 32 × 32 × 1 is used for the initial convolutional layer. Results are retrieved on the Malimg dataset where 25 families of malware are fed as input and our model has detected is Alueron.gen!J malware. The proposed model CNN-DMA is 99% accurate and it is validated with state-of-the-art techniques.

## 1. Introduction

We are living in a time where technology is rapidly progressing, i.e., IoT and 5G. The telecommunication sector has already completed the four generations and the evolution of 5G is the latest network, whereas the future contains 6G. 5G is highly supported by the wireless world wide web having features such as high speed, high capacity, and large broadcasting of data in gigabytes per second. Multimedia newspaper and television programs have become more effective and attractive with high definition [1]. The upcoming 5G network has offered several basic services such as massive machine-type communication, enhanced mobile broadband, and ultra-reliable low-latency communication. With the mixing of new applications and technologies, now 5G works on the “Internet of Everything”. In the 5th Generation network, many different mobile devices are connected: few applications in 5G Internet of things (IoT) is an industrial Internet of things (IIOT), Smart Agriculture, Intelligent Transportation System (ITS), Smart healthcare systems [2], and Smart Home and Mobile Caching [3].

Healthcare is now an important emerging field because of the COVID-19 pandemic. By integrating healthcare and 5G, the 5th generation has low latency and high computation contributes to Wireless Tele-Surgery (WTS), where surgeons operate using the robot at distant locations is the first use case here [4]. Due to the great development in technology, there exist different requirements that can be fulfilled by 5G. However, there exist some limitations.

Practically, 5G-IoT provides the environment to make information and communication secure and private so that attackers cannot access the secret information of the patient [5,6]. As IoT is an integration of different connected devices, it is prone to vulnerabilities and security threats. Several techniques are working on the security threats of healthcare systems like Machine learning, Deep learning, fog-based, cloud-based, IoT-cloud-based, and blockchain. With the use of fog computing and edge computing the parameters such as response time, energy efficiency, and cost can be managed [7]. To maintain data integrity, one of the techniques called blockchain and encryption for secure communication is being used [8].

For instance, technologies such as fog computing, edge computing, and blockchain are moving towards increasing potential development in Machine learning (ML) and Deep Learning (DL), both of which can produce accurate outcomes [9]. Despite supporting all these parameters, both ML and DL can also play an important role in the healthcare field for maintaining security in several types of attacks, and we are shedding some light on one of the attacks known as a malware attack.

### Our Contributions

1. The different generations of networks of the healthcare system have been discussed in the paper. To propose a new security model for healthcare and analysis of previous models are the key points of the article.
2. 5G-IoT plays a major role in providing the fast network to conduct various operations in the field of healthcare applications where sensitive data is stored at a remote location, thus layers of 5G-IoT are vulnerable to attacks. A variety of different types of attacks along with some of the possible solutions are proposed.
3. Among various security threats to the 5G-IoT Healthcare, malware is the most prominent. The creation of millions of malware files destroys the fields related to healthcare, the Internet of Things, social media, banking, intelligent transportation, smart homes, etc. It becomes crucial to detect this attack, which is done with deep learning.
4. A deep learning CNN model is proposed for the malware detection (CNN-DMA) in an image whose input is in the binary form which is converted into grayscale and then among 25 families of one malware attack is detected with an accuracy of 99%.

## 2. Generations and Challenges of Healthcare Systems

**Figure 1:**
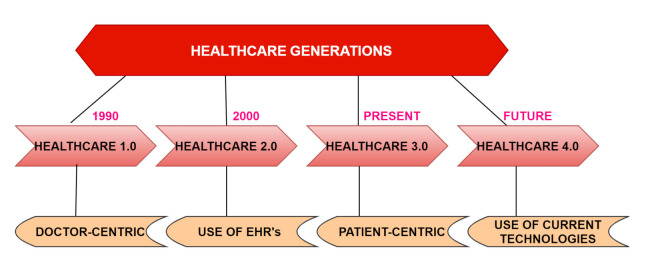
Generations of healthcare.

Step by step updates in the previous technology give rise to the next generation. The Healthcare field is also progressing with the technology change. The four generations of healthcare are shown in Figure 1 and are discussed as follows.

- *Healthcare 1.0:* It is a traditional method of healthcare. It was initiated in the 1990s, where patients suffering from any illness go to the clinical center to meet with the doctor for treatment. Then, the doctor will diagnose the patient based on a generated report after testing the patient, where the medical history of the patient is kept paper. A doctor’s medical knowledge can be judged by his/her diagnoses and what is given to the patients.
- *Healthcare 2.0:* The next version is Healthcare 2.0 of the 2000s where all doctors, patients, caregivers collaborate, where the medical history involves social networking in which each patient can participate. The best part of this is that patient is involved in their own healthcare decision. This also includes the data analytics between the patient and the physician as the patient can share his/her electronic health records (EHRs) with the doctor and medical researchers. It is easily affordable for the patients and provides quality care.
- *Healthcare 3.0:* The current healthcare system, in which organization of EHRs is conducted for the creation of an Open System so that healthcare facilities can be easily accessed by all. The use of virtual tools in such a way made interaction between doctors and patients much better. In Healthcare 3.0, two technologies play a vital role: information and communication. The motive behind this particular vision is that the experience and capabilities of doctors must be utilized and the administrative burden should be lessened [10].
- *Healthcare 4.0:* It is the next future version of healthcare introducing augmented and virtual reality. EHR repositories are being used to share the patient’s health information among the doctors anytime and anywhere. The best part of this version of the healthcare system is that the patient’s health records are being shared among different doctors so that the best medication, diagnosis, and treatment can be given to a patient. However, there are problems with data sharing as it affects data security, authenticity and authorization of data, secure communication, etc. This system also supports health assistance through applications and websites.

### 2.1. Challenges of Healthcare

There are various factors affecting healthcare systems that make the implementation of modern techniques difficult [11,12]:

- *Non-Patient-Centered:* It is not good for those patients who have some significant illness such a case when a patient needs to go to a doctor but he/she is not in a position to go as he is prescribed to take some rest. At the same time, a supervisor of a patient needs to schedule an appointment in their busy day-to-day life before visiting a doctor.
- *Non-customized:* A doctor’s prescription for an individual patient is not based upon the type of problem he/she is suffering from but based on the population average that might not fit for the patient. These days treatment for any individual illness or disease is costly and non-affordable for lower and middle-class people.
- *Non-accessible:* Disabled patients who cannot visit the hospital alone cannot use some facilities, and thus these facilities may only be utilized by few groups of patients. That is why limited access to basic facilities is a risk to many patients.
- *Huge Connectivity:* IoT is a connection of billions of different devices together in the network to produce required information, and it is important to give guaranteed connectivity and high mobility to the devices connected in healthcare like fast ambulance facilities and immediate emergency treatment to the patient.
- *Power and Cost:* In IoT, large number of devices and sensors are connected incurring a high cost. Operating a large number of devices and sensors consumes a large amount of power, so in healthcare achieving low power and low cost despite having many connections is a big challenge.
- *Security and Privacy:* Both of these parameters are important in every field like smart home, smart transportation, healthcare, and more. Various heterogeneous devices are connected in IoT, therefore security is a major concern. Sometimes the IoT architecture lacks private communication, data integrity, and authenticity, making the healthcare domain more vulnerable to attacks [12]. The use of IoT in communication between healthcare devices and the cloud must be secure to make people more confident in e-health services. However, less risk should be there to prevent the system from new attacks [13].

### 2.2. 5G-IoT for Healthcare: Applications

With the increase in population, most of the active individuals are busy with their daily life routine, and as such they are unable to monitor and track their health records on daily basis. Usually, people visit the doctors when they feel any health-related problem at a higher level, which is sometimes too late to cure. There are some countries that are providing programs for adopting a healthy lifestyle and maintaining good health [14]. Therefore, low-cost and efficient health-related facilities must be given to a patient such as a doctor’s proper assistance, secure emergency treatment to the patient, and ambulance facilities must be provided.

The work in [15] characterizes a telemedicine system that can handle the cases remotely; a telemonitoring system is installed at the patient’s home and the doctor can monitor from the office only. Telemedicine can be done in either a synchronous or an asynchronous way. Synchronous telemedicine needs a link so that doctors and patients can communicate with each other, i.e., a face-to-face interaction using any application such as secure video calling. However, in asynchronous telemedicine, there is no need for a communication link as the required information inquired by the patient will be securely transmitted by a doctor. Some of the main services offered by the telemonitoring system: First, it provides safety as an important factor as installation of many sensors can detect what is happening in the environment. Another service provided by 5G is localization when the needed system provides the location of the user. Third is the interaction by name, which gives important information, the medium of communication, contact management, etc. Fourth is the real-time alert, as it is used to send alerts for patient relatives and caretakers. The fifth is the training part, as this application allows doctors to supervise patients and access patients’ important information from anywhere [16].

Security is an important factor as we are living in the era of hackers. Important and sensitive data are always vulnerable to attacks as there is an almost 100% chance of data being lost. Therefore, one must ensure that the transmission of data, storage of data, and processing of data must be done in a secure manner. If the patient’s health-related data are being stored on a cloud server, then there is a chance that the patient loses control of their data. Encryption of data before uploading is mandatory. Therefore, security and privacy here play an important role. There are some surveys (discussed below) in which the authors dealt with the security and privacy issues, e.g., Ermakova and Fabian (2013) came up with the secret sharing with no link between patient and medical record as confidentiality must be maintained by assigning patient with the identifier. However, the problem that arises here is that in the case of an emergency, how are medical staff to identify the patient in the absence of an identifier. Li et al. (2010) gave a solution to this problem by making a model in which the patient can provide only important related information to the staff and can hide the remaining.

In [17], a comprehensive survey contains the limitations of the current technology (cost, latency, and data transfer rates) in healthcare applications and the use of WSN for tracking intake of medication and monitoring daily activities that can be overcome by upcoming fifth-generation remote access. The survey in [5] consists of the use case WTS where the doctor is located far away and can operate with the help of robotics considering parameters like stability, reliability, speed, latency, throughput, end to end delay, bandwidth, security, long battery time, and network capacity. So far, the current technology cannot fulfill these networking parameters. The coming 5G provides a wider perspective in terms of those parameters. The surveys in [18,19] concluded that 5G, being reliable, can handle voluminous data having three properties—embb, urllc, and umtc—that allow tele-consulting and telesurgery applications to work. Working of WTS is just like Mater–Slave system where a Human surface interface (HSI) having a video console, headphones, and haptic device at the master side can operate on the patient using a robot by giving certain commands. The robot for performing telesurgery is provided with the a 3D camera, some sensors, and a microphone and comes under the slave side [20]. This WTS network is also vulnerable to attacks such as DOS, MITM, and MALWARE attacks. Due to all these attacks, robots can be affected at the time of performing surgery, which may put the patient’s life at risk. The use of various cryptographic algorithms is a solution to all these problems, but the complexity of these algorithms must be reduced. There are some techniques like Parity check, lattice coding which will not able affect the WTS performance.

The authors of [21] identified the parameters discussed above which are required to implement m-health which is a mobile health application. This mobile app would be able to access different people like patients, doctors, and nurses, and give solutions according to the requirements. In our current rapidly developing world, everybody needs everything in their hands, and nowadays the population’s hands carry a small object that is a mobile phone which is now everyone’s basic need. From calling to sending messages, playing games to video calling, surfing the internet to watching TV, and studying to banking everything is possible from just a small device. If we talk about the healthcare field, here mobile phones also play a crucial role from tracking daily walking steps to monitoring pulse rate, tracking how many glasses of water intake, and more [22]. Discussion of some security services for security management in mobile devices follows.

- *Data Communication and Storage:* Data must be encrypted for strong communication between the patient and healthcare provider so that intruders cannot intrude into the network.
- *Policy Guidelines:* Whatever technology is being used policy guidelines should be prior declared, e.g., restricting user access must be informed before the policy violation and management of wireless network interface should be under policy guidelines.
- *App Installation:* This involves trusted entities that would make sure the applications we are installing are trustworthy by verifying them with the digital signature [23].

Mapp et al. [24] consider some solutions to manage mHealth data using cloud computing techniques.

- *Encrypted Data:* The mHealth data must be stored and the packets transmitted must be in an encrypted manner. Encryption is mandatory all the time except for when data are urgently needed [25]. As it is an application, the organization who owns the particular app can be given access to decrypt the data.
- *Confidentiality:* It must be maintained so that the unauthorized user cannot access the patient’s sensitive health data; only the authorized user can have access, read, modify the patient’s data [26]
- *Authorization:* Only the authorized user has the right to access the private health data of a patient [27].

Another application is the online health monitoring discussed in [28] which comes with an affordable and reliable device that is secured in storing data on the server-side and data can be monitored using medical sensors. In case of a health problem, the device alerts the registered doctor as well as the patient. The extension to this is to create a type of model in which any doctor or health expert can access patients’ data [29]. When using the agent and manager model, where the agent collects the signal and sends it to the manager through web interface handover to a healthcare provider, secure communication is a strict requirement. For remote diagnosis, i.e., another application offered by healthcare facilities in [30], while the survey has been conducted for the identification of various requirements such as low latency, enhanced Mobile Broadband (eMBB) required for the expertise in telediagnostic tools, remote access to the robot, and haptic feedback [31,32].

The survey in [33] discusses Augmented Reality (AR) and Virtual Reality (VR). AR is assisted to give the type of disease and the required information to a surgeon for any surgery whereas VR trains the professionals to perform surgeries. The 5G healthcare applications with some advantages and disadvantages are being discussed in Table 1.

### 2.3. Security Issues of 5G-IoT Healthcare

The 5G network has the following security risks.

- *Large connectivity of 5G network:* The large-scale connectivity of the 5G network leads to distributed denial-of-service (DDOS) attacks. 5G technology supports huge connectivity, i.e., 1 million connections/km and hence vulnerable to attacks that launch huge traffic at the same time and affects the network capability.
- *Low latency and high bandwidth:* These characteristics can put the security at stake which may increase the difficulty of security protection, cryptography (encryption and decryption), content identification. 5G’s ultra-reliable low latency feature required the strong need for network security, malicious traffic attack prevention, ability to encrypt and decrypt transmitted data that automatically increases the need for network security.
- *D2D and edge cloud communication:* Introduction of both these changed the original architecture and modes of communication [34] that has more impact on content security as compared to the centralized server. It makes the centralized monitoring system ineffective. Therefore, it made traffic security difficult in edge cloud service and D2D communication.

**Figure 2:**
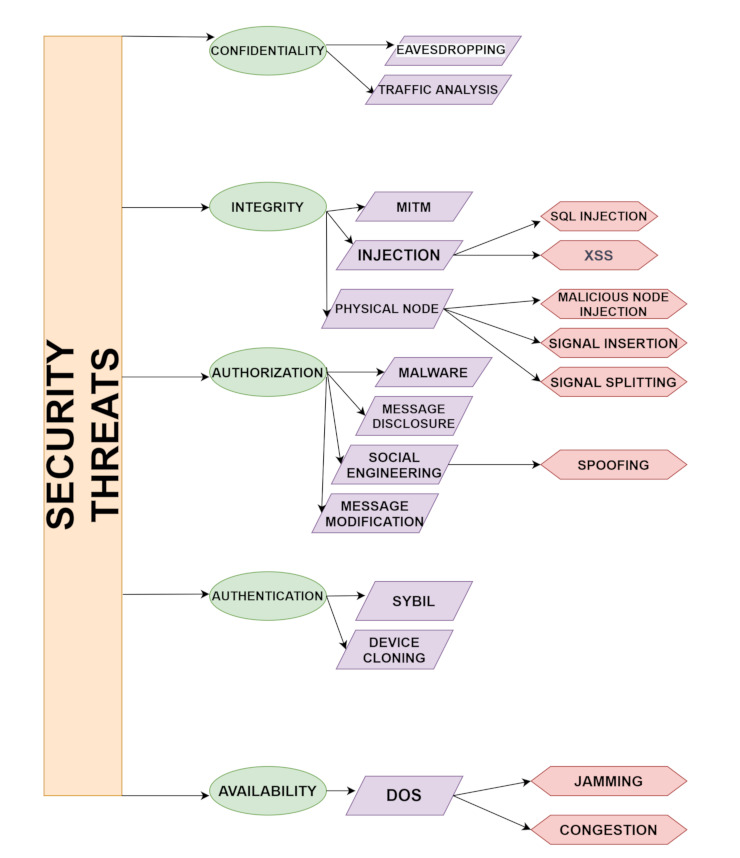
Security threats with types of attacks.

Growth in technology does not led to secure applications every time. The same is to be seen in the domain of healthcare which also has some security threats that need to be discussed [35,36]. Some of the security threats are mentioned in Figure 2.

- *The risk to patients’ private health records and theft of medical data needs to be monitored on the large scale:* There exist wide applications of 5G as discussed above which involve the patient’s information and it is required to be private such as EHRs, laboratory data, and medical-related images. The leakage of sensitive health records of any patient will automatically lead to one of the threats to healthcare.
- *Attacks on the 5G healthcare network:* 5G healthcare applications, including remote surgery and emergency treatment, lead to the requirement of reliability and security to avoid transmission delay in the 5G network. In case of the security attacks on the infrastructure of the applications lead to a serious impact on the patient’s health and even the death of the patient.
- *Malicious attacks on the records:* Various capabilities of 5G made the collection of medical data possible on a large scale, from different sources as well. Therefore, the collected data can be used to detect and outline public health-related events, i.e., unknown diseases in a punctual manner and epidemics. Due to the collection of huge data in one place, data manipulations in medical records are possible by the attacker which leads to distortion, sometimes resulting in failure of the emergency response mechanisms. Note here that the threats discussed above can be from both external and internal networks.

## 3. Security Issues in Healthcare

As the healthcare system contains sensitive information and makes use of data sharing, data storing and communication takes place via sensors in-body, off-body, or on-body. Therefore, making the system more efficient, reliability and data security are the most important challenges to deal with [37]. There are privacy issues and security threats to the 5G-IoT Healthcare system. Attacks could either be Active where the attacker attacks the network and changes the original data or passive in which the attacker aims to obtain valuable information but cannot change the original data that results in confidentiality, access control, integrity, authentication, and availability attacks [38]. Attacks that correspond to the confidentiality of the system are eavesdropping and Man-in-the-middle. Attacks like Replay, Injection in data, and Denial-of-Service (DoS) attacks affect integrity. Authentication deals with jamming and flooding attacks. Some malicious attackers use extreme ways to steal sensitive information. By keeping all this in mind, we can see maintaining privacy in healthcare is a very important aspect to take care of. This means that the patient’s choice to share their data is of high importance. It should be in a way that patient’s data should be shared within the system network and with the professional staff and some sensitive information should be kept secret. Therefore, by using smart applications or the smart environment’s sensitive data, personal information is more vulnerable to attacks as it is more prone to security threats. In Access control, attacks sometimes violate data security or sometimes specific policies are being applied. PASH (Privacy-Aware S-Health access control system) is a Ciphertext-Policy Attribute-Based Encryption (CP-ABE) scheme that was introduced in [39], where encrypted s-health records(SHR) hide access control policies and attribute values can only be seen. To make the technique efficient the decryption test was done with fewer bilinear pairings. Theoretically and Experimentally PASH is secure and effective as compared to other techniques. Software implementations for medical sensors is also a challenging task as developers in case of wireless connectivity will be in more trouble in case of security and privacy.

- *Confidentiality:* Protecting confidential data is the major issue. In healthcare systems, WBAN nodes are considered important as they contain the private information of patients therefore protection of data is a must and must be protected from unauthorized access. At the time of transmission, vulnerable data is a huge overhead that damages the network and the patient’s trust. The best solution to this is the use of encryption between WBAN and the coordinators [40].
- *Integrity:* To protect any packet’s content and its accuracy, its integrity must be maintained. The problem of external modification is not solved by data confidentiality as modifications are easily being done when integrating message fragments, changes made in data within the packet, and even at the time of sending message fragments. In the healthcare system, it is a big issue as modifications in patients’ health-related status that can even lead to death in some cases.
- *Authentication:* Whether it is a healthcare system or any other field or application data authentication is a must requirement. Therefore, the nodes having information and part of wireless body area networks must be knowing which is trustworthy and which is not.

### 3.1. Attacks on 5G-IoT Healthcare Layers

**Figure 3:**
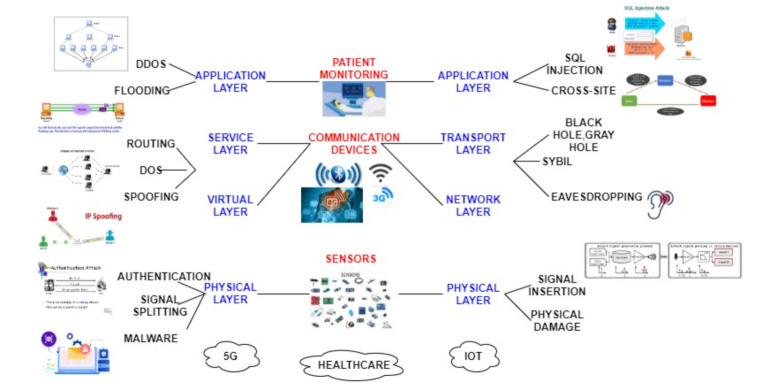
5G-IoT with possible attacks on the layers.

As 5G-IOT’s integration with the healthcare system is vulnerable to attacks. With the combination of the 5th generation network and IoT devices, healthcare, and other applications can work smartly. Formally IoT is “a network of items and each item contains sensors which are connected to the internet” [41]. Improvement in healthcare surely is seen with the application of 5G-IoT. To view the big picture, the architecture of IoT in 5G will be discussed which consists of different layers namely the Sensor layer, Network layer, Communication layer, and Application layer [42]. The details of possible attacks on layers along with the elaboration are discussed in Figure 2 and Figure 3.

1. *Physical Layer:* This is the physical layer having sensors, actuators, controllers, and devices that are used for communication with the next layer. To reduce power consumption and increase computation power, some small devices like Nano-chips are being used which produce huge processed data [43]. There are different types of e-health sensors which means the connection of the biosensors and IoT enabled healthcare such as clinical diagnostics, cardiac activity monitoring, sleep monitoring, woman health monitoring, infant monitoring, continuous glucose monitoring, fitness tracking, etc.
2. *Network-Virtual Layer:* Works on the principle of LoRAWAN have low power, and works on the central server have high communication and connectivity. IoT system’s efficiency can be increased from the heterogeneous devices used that communicate with each other. To improve the D2D communication 5G is the technology that is being used to provide better connectivity for Machine type communications.
3. *Transport-Service Layer:* Information is being transferred from the following layer as this is the heart of the architecture. Without this, no network can communicate properly.
4. *Application Layer:* The application layer uses network integration of all devices and sensors. As the name suggests containing all the IoT smart applications like smart cities, smart industries, smart homes, etc.
  Low latency must be provided as it should be considered important because it is the transmission time to transmit the information in the packets between sensors and processing unit by speeding the transmission up.

Here are the possible attacks that take place on the above-discussed four layers of 5G-IoT-Healthcare:

- *Signal insertion attacks:* To degrade the services, the quality of connections is affected and attacked by feeding damaging signals inside the network.
- *Signal splitting attacks:* It refers to breaking of the communication and getting rid of the signal in a network so that attacks like eavesdropping could happen where an intruder can get access or listen to a conversation secretly or signal can degrade.
- *Spoofing attack:* In the existing network, an intruder can intrude with the help of a copy of each packet that affects confidentiality and privacy, which is a must in a healthcare system [44]. In a sensor spoofing attack, the physical environment is altered in a way that means the medical system cannot work properly [45]. In [46], a sensor spoofing attack against the infrared drop (ID) sensor was introduced in an infusion pump.
- *Single component attack:* This attack can take place by damaging a switch or breaks down fiber by cutting or any other means which lead to component failure. As in the case of wireless telesurgery, attackers can attack the healthcare network system while operating robotic surgery which can be harmful to the patient’s life.
- *Distributed Denial of Service:* It enables the victim to use the resources and affects network connectivity by flooding of messages. In the case of healthcare, an attacker can interrupt the patient’s side while obtaining the information online.
- *SQL Injections:* It is a structured query language through which attackers can hack the database and personal information can be stolen. For example, the patient’s database containing health-related information is maintained so that it can be attacked by the attacker for obtaining that patient’s information.
- *Cross-site scripting:* An attack in which malicious code is attached to the victim’s browser resulting in stealing passwords, cookies, etc. [2]. This attack can be more dangerous at the time of payment where account details including passwords can be fetched by the patient.
- *Routing attacks:* The attacker modifies the route of packet transmission from sender to the receiver so that the packet cannot travel from source to destination.
- *Message disclosure:* In this attack, the patient’s log files access rights are breached and sensitive information is stolen by an attacker.
- *Message modification:* A message traveling from a patient to a doctor is modified by an attacker which can harm the patients because of the wrong treatment based on the wrong diagnosis from the alterations made by the intruder.
- *Eavesdropping:* Through an open smart healthcare system, the intruder listens to all the sensitive information provided [47].
- *Replaying attack:* After eavesdropping, modified information is forwarded by the intruder. The survey in [48] introduced One-touch Ping insulin pumps and blood glucose meters that allow attackers to record the transmission and replay them later that do not use any timestamp.
- *Compromised node attack:* In this type of attack, the attacker injects false data by attacking a particular node.
- *Denial of service (DoS) attack:* This attack involves the flooding of so many requests at the same time to a server generates so much traffic so that server cannot be able to respond to the particular request [49].
- *Black and gray hole attack:* A network is affected by putting an infected node that changes the entries in a routing table containing information of all of its neighboring nodes and the information is being sent to the compromised node. Both Blackhole and Gray hole attacks can be differentiated, as gray hole attacks reply with some data that is non critical to its neighboring nodes whereas black hole attacks do not reply.
- *Sybil attack:* These attacks modify the entries in a routing table by using an infected sensor that impersonates multiple sensors.
- *Social engineering:* This type of attack exploits the user in such a way that they share their personal information with the attacker which would benefit the attacker [50].
- *Traffic analysis:* In this type of attack, intruders just want the information from the characteristics such as locations of both sender and receiver intruder observes and no manipulation of data takes place [44]. All the possible attacks on healthcare systems are being discussed above.

### 3.2. Malware Attack

Malicious software is designed in such a way that if given access to other computer systems it performs harmful operations to obtain personal information. Various types of malware exist, such as viruses, worms, Trojan horses, advertising software, spyware, blackmail software, etc., just to obtain personal data, system hijack, identity stealing, monitoring users are the big threats to operating systems and users. Malware has 25 families, and is gradually increasing. Therefore, it is very challenging to stop malware attacks, as all the personal data of users as well as industries or companies are at stake [45]. The healthcare domain is facing more problems related to malware attacks as in this sector the security plays an important role. Globally it is a more targeted sector. Increasing variety in the upcoming generation networks services and devices, verification of security policy in various autonomous decision making, and subsequent implementation of the network gave rise to DL. Various efficient malware detection frameworks for Android smartphones are used in networks, based on the deep neural network called DLAMD for rapid detection and the deep detection phase of malware attack. Deep Learning also helps in working in network operators in the absence of previous data or experiences or difficulty in understanding the data with classical approaches. Without using DL it is very difficult and unmanageable to practically differentiate between a security attack from legitimate traffic. Machine Learning also plays an important role in avoiding service interruptions by identifying terminal actions and requirements. In network security, the basic use of DL is to recognize malicious traffic (malware). Several types of malware are made with functionalities such as bottleneck, gain access to the root, malicious program downloaded through the third party, location-related information being stolen, and acts as Trojan. Some malware is being sent to a remote server and some are remotely controlled by the server-side. Furthermore, DL helps in the threats posed by ransomware. Ransomware is a type of malware that makes the user unable to use its system, files, or operating system [46]. It is very difficult to recover from ransomware as there are a lot of encryption techniques that are being used. The rapid increase in AI collaboration with DL gives the best results against ransomware thus making the system more secure. DL also used for the detection of ransomware possess more interest because both of them can be able to detect zero-day threats. Some of the security threats with the possible solutions and requirements in some of the healthcare applications are shown in Table 2.

## 4. Deep Learning in Secure Healthcare

**Figure 4:**
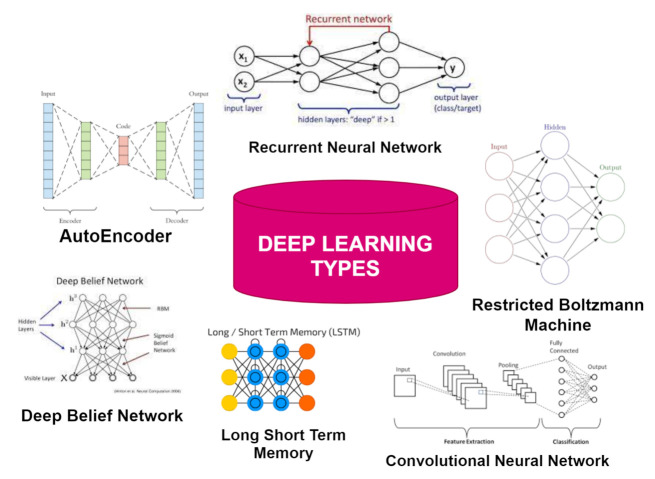
Types of deep learning.

**Figure 5:**
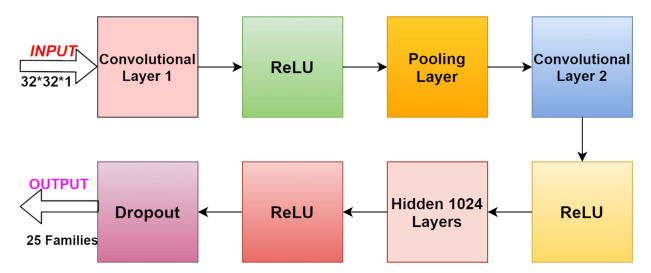
Convolutional neural network architecture.

As far as the advancement in technology is concerned, computers have already shown the best results compared to human beings in various fields such as games, voice recognition, image recognition, etc. Through deep learning (DL) it is possible for computers to achieve those capabilities similar to human beings [53]. The various types of DL classifiers are shown in Figure 4 and Figure 5. In DL, computers can learn based on past experiences, and from that experience, a training model is built without any prior knowledge and useful patterns will be extracted from the raw data. DL has much power when the available training data are large in number. Among the neural nodes, weights are determined by the computer through a training process and input data is extracted. After completion of the training process of neural networks, to achieve a reward a proper decision is being made. The successful results of the above process can be seen in many real-time scenes such as games [54], voice and image recognition [55], drug discovery [56], etc. For a better quality of service and experience that can be achieved by DL for network nodes in which features can be obtained with the representation of complex network scenarios.

DL can solve several complex problems in computational time using a device that enhances the operation of one or more subsystem’s speeds [57]. Extraction of well-represented features is quite difficult using Machine Learning algorithms because of the following constraints: known specific domain, required expert knowledge, and computational bottleneck [57]. Prediction Accuracy Parameters like mobility, channel interference, and variation are not being analyzed in ML but can be possible through DL as it has deep neural layers. Therefore, patterns hide in the input can be extracted from one layer first then from the other layer with the help of DL algorithms thus supporting high accuracy and pre-processing of input data. The need for preprocessing of input data is available in ML only and not in DL because the input of DL is feature parameters that can be integrated from the network only.

The Deep Learning approach is used in several fields applications of DL are vision, bioinformatics, natural language processing, etc. [52]. In [58], a great improvement is being achieved in the performance of DL over ML. The applications of DL discussed above need data in large volumes so that the data can be used to transfer over the network therefore DL achieves a great performance level than ML. One more advantage of DL over ML is without any human interaction and to give a solution to any problem DL works with the new features. There are two neural networks commonly used which are Convolutional neural networks (CNNs) and recurrent neural networks (RNNs).

CNN allows difficult operations on the hidden layers to reduce the parameters and share weights. The local information can be extracted from a table-like structure (grid) input data. The variable-length sequential input data are processed by RNN and output can be produced at each step. Based on the current input data and the previous step’s hidden neurons, hidden neurons at each time step are calculated. Long short-term memory (LSTM) is an artificial RNN with feedback connections that process an entire sequence of data.

### 4.1. Types of Deep Learning Handling the Attacks in Healthcare

Though DL is used in various applications, providing security here is a major concern. As a result, this section discusses the handling of security attacks using Deep Learning. The survey in [59] introduced a learning-based Deep Q-Network approach for resolving the authentication, malware detection, and access control issues. In [60], an intrusion detection system for an IoT is proposed and the environment is designed and implemented for attacks like spoofing and sinkhole, and also detects all the implemented malicious nodes with minimum overhead, limited energy, and memory capacity. The survey in [61] focused on IoT security and privacy features related to the healthcare field with the required security necessities, threat models, attack classification also proposed an intelligent security model for minimal security risk. In [62], the use of the two types of DL algorithms, namely, Convolutional Neural Networks (CNNs) and Deep Neural Networks (DNNs), for building a type of model in which IoT hardware that works in smartphones and wearable devices (wearable cameras, fitness trackers, and smart jewelry) can process sensor data. The survey in [63] uses long short-term memory (LSTM) for handling authentication in IoT environments. The authors have presented a comprehensive and illustrative survey on cutting-edge and intelligent IoT healthcare systems for the classification and prediction of healthcare data using edge intelligence. They have focused on research articles on Edge and IoT published between 2016 and 2020, and also discussed solutions for issues related to cloud and edge computing, AI, IoT, and various types of medical signals. The current challenges and future directions in these areas have also been addressed by the authors.

The design of LSTM is sensitive as it is designed only for device authentication and cannot detect any other types of attacks. The survey in [64] uses LSTM for botnet detection in IoT. To manage authentication, access control, and intermediate attacks in IoT, the survey uses a deep learning-based deep Q-network to maintain security and privacy. The data in the healthcare field are increasing rapidly; therefore, it is mandatory to protect this large amount of healthcare data, effective solutions must be taken. To secure wireless communication, secure communication cryptographic solutions are needed. The following symmetric [65] and lightweight [66] cryptographic protocols provide the solution for access control in healthcare and protection against spoofing. Integrating cryptographic solutions with the current healthcare devices like Implantable Medical Devices must be designed again for compatibility but it would be difficult in an emergency case where there would arise a need to communicate with unauthorized professionals but that can be interrupted due to cryptographic mechanism. Therefore, there is a need to focus on cryptographic solutions that would be medical-centric to meet the security needs of the healthcare-based system. The survey in [67] came up with the connected healthcare systems (CHSs) that are used for remote monitoring of patients and elder’s physical conditions and this system is vulnerable to attacks like eavesdropping and relay attacks. The basic reason for the security of healthcare devices is to ensure the safety of patients’ life. An IDS using stack autoencoder is used to detect any type of attack in which an alert is generated to aware the staff and patients that the attack is about to happen on the healthcare device. External devices generate incoming traffic to the healthcare devices based on the parameters like length of the message payload, the difference between successive requests from that of the peripheral devices [68]. Therefore, there would be more focus on developing IDS with those of the parameters keeping in mind that can be compatible with the communication protocol standards in healthcare devices.

First, intrusion detection systems (IDS) were introduced in 1986 by Denning et al. [67] for the recognition of attacks in an IoT network. An intrusion detection method using deep learning’s stacked autoencoder (SAE) designed for security and privacy. However, there exists a flaw between both deep learning and the lightweight intrusion detection (ID) method. Detection efficiency or rate can be improved using ID without any increase in workload. The survey [51] designed an intelligent intrusion detection system with the help of DL algorithms that uses Deep Belief Network (DBN) to make up the Deep Neural Network (DNN) as a learning model and integrates virtual network with those of the DL algorithm to detect malicious traffic. The result of which is the optimal solution for DL-based IDS for blackhole attacks, DDoS attacks, sinkhole, and wormhole attacks with both precision and recall rate. In the future, this system can be expanded for spoofing and Sybil attacks. The survey in [37] focuses on IoT communication standards and technologies, and energy-efficient IoT-based healthcare, privacy, and security challenges in healthcare. For future perspective, the use of a new type of sensor and advanced communication technologies parameters like reliability, resource management, network coverage, etc. will be utilized. The survey [69] proposed an Intrusion Detection System (IDS), HEKA, in which Personal Medical Devices (PMD) will be monitored and several attacks like eavesdropping, man-in-the-middle, replay, false data injection, and denial-of-service attacks would be detected with accuracy. The survey in [60] discusses privacy and security threats and consequences of those threats that affect the healthcare system as well as security measures and limitations for healthcare systems. Future works must focus on the security of healthcare systems against vulnerabilities. The survey in [70] discusses threats, vulnerabilities, and consequences of cyber attacks in the healthcare system. The comparison table of accuracies with the others insecurity is being shown in Table 3.

### 4.2. Deep Learning’s Convolutional Neural Network Classifier in Secure Healthcare for Detection of Malware (CNN-DMA)

*Convolutional Neural Network:* The structure of a CNN is composed of three parameters: dataset size, quality, and type. The input layer’s portions can be processed by the multiple receptive layers. These networks can be arranged in a way that overlapping of input area can be created to obtain the output of a high resolution of the original image. CNNs use operations such as pooling and convolutions for feature detection. After feature extraction, all fully connected layers work as a classifier. The coupling of convolutional layers with pooling layers is less as it is non-compulsory for the CNN to be fully connected. It is considered the heart of CNN. The use of convolution is in the combination of two mathematical functions the result of which is also a function. The execution of convolution is done over the input by sliding the filter. For every location, matrix multiplication is performed and the result is summed up onto the feature map. This layer has p*p*s input image where p is the height and width of the image and s is a channel with varying filters and the size of each is q*q*t. q is smaller than the image dimension and t could be the same as channel s.

- *Pooling Layer:* The addition of a pooling layer between the CNN and after the convolution layer takes place. Reduction of dimensionality to achieve low computation and less number of parameters is the main goal of this layer. The most important pooling is max pooling. It is used to pick up the maximum value in each window.
- *Fully Connected Layer:* The last layer after convolutional and pooling is the Fully Connected Layer to classify input images. In a fully connected layer, having neurons that are connected to the previous layer activation functions. The following section will work upon how artificial neural networks or deep learning classifier convolutional neural networks for detecting malware attacks as the network 5G, IoT, healthcare domain is vulnerable to many attacks. Along with the detection of malware, it will also provide the security of information to detect and classify malicious code.
  Though every organization contains personal information or data and that information or data both are vulnerable to so many attacks as in the healthcare field patients’ life will be at stake. As a result, many attackers or criminals gain new techniques every time to attack the target. Many ways are being used by the security vendors to defend against these types of attacks but are unable to because of the billions of malware discovered on the monthly basis, and it is impossible to achieve that. Therefore, approaches like deep learning are a must to provide security and privacy. The architecture of CNN is shown in Figure 5 where the input will be an image of size 32*32*1 fed to the convolutional layer 1 then to the rectified linear unit

## 5. Results and Analysis

### 5.1. Malware Detection and Family Classification

**Figure 6:**
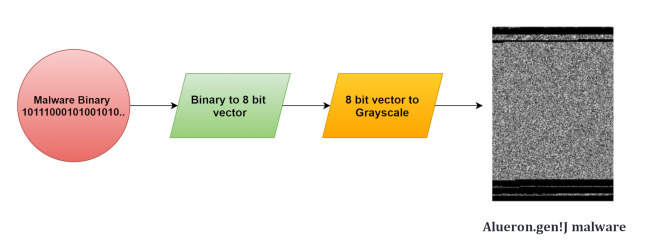
Conversion of malware into image.

**Figure 7:**
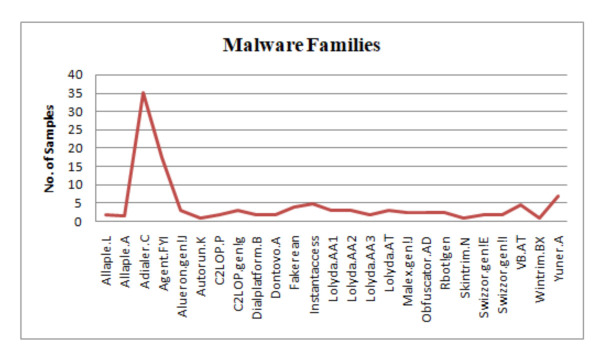
Malware families.

**Figure 8:**
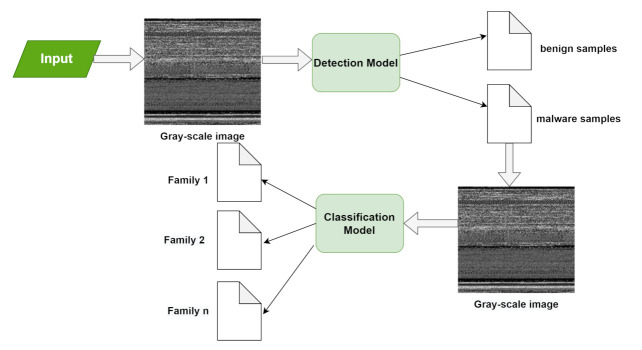
Malware detection and classification.

The dataset which is being used here is malimg with various parameters. The training of the model is done by reducing the size of an image in the following dataset. The problem that arises here is the small size of an image that cannot handle all the related information as there are high chances of loss of information and the higher value will only complicate the calculation results; it is also unable to improve the accuracy. Figure 6 converts the malware into an image. We use the Malimg dataset, which is a large-scale unbalanced malware with a total of 25 malware families, as shown in Figure 7, along with that of the 9339 malware grayscale samples including Backdoor, Worms, Trojan horse, and Rogues software. First, the dataset is divided into 60% of the training set and 40% of the testing test. The training set is used for the training of the model and the testing set is used to check the performance of the model. The experiment took place on the TensorFlow2.0. The working of the model depends on how well the model is performing based on parameters such as accuracy, recall, and F1 score so that the best model can be used for detecting malware. The malware detection and classification is shown in Figure 8. An input is given which can be converted into grayscale image the detection model then detect the attack and divide the samples into benign sample. A grayscale image then obtained on which classification model is applied for obtaining the malware families. As there exists an imbalance between the various malware classes only accuracy is the parameter for the model’s overall prediction. The small number of classes ignores the ability of prediction. However, if there exists an error in a small number of classes, then there is a chance of having high classification accuracy. The results will give a confusion matrix of 25*25 along with the following parameters: precision and recall. The performance indicators are responsible for achieving better classification also lower misclassification. The malware detection experiment is conducted on a Malimg dataset. The use of dropout layer is used to prevent the overfitting of the model. As the conclusion is the dropout layer is responsible for the detection as the wrong predicted samples can be predicted. This particular dataset has many samples with many obfuscation techniques such as encryption and packaging. Some of the encryptive malware families includes Autorun.K, Allaple.A, and Allaple.L, which are used for the encryption of several layers of code part with the use of a random key. Furthermore, the same family variants include Swizzor.gen!E and Swizzor.gen!I along with Yuner.A, Malex.gen!J, and Rbot!gen. Before discussing further, there exist some steps to load the data where an introduction to the layers used in the model and feature extraction is a must.

### 5.2. Steps to Load Data

The following model can be achieved by performing on Raspberry Pi3 with 4*ARM Cortex A-53, 1.2 GHz processor, and 4 GB RAM. The model for human activity recognition is implemented in Python 3.8.8 and Keras. The building of the model is done by Keras using layers of Dense, Dropout, and Flatten. The dense layer is used to change the vector’s dimensions. This layer also operates on the parameters such as rotation, scaling, and translation on the vector. Then, the dropout layer makes the network learn the features having different subsets of the other related images. The dropout feature is also used to double the loops for the combination. The result of which is the training time for each epoch is less. The hyperparameters are optimized, which are shown below after that the training of the model takes place using final parameters such as epoch is 20, the batch size is taken to be 64. And number of classes to be taken are 25 so that the detection of malware can be analyzed. Refer to Table 4.

### 5.3. Feature Extraction

The most important step is the feature extraction part in which the data size is very much large and difficult to manage. The next step is the data transformation into some reduced set of feature representations. A feature includes various parameters such as the size of data, color, image’s shape, etc. [71]. Here, some of the local features, global features, and texture features are being used. In the following framework, the two stages are being performed with the use of an equivalent stream. The first stage consists of the texture feature extraction from the grayscale images because of its low cost of computation and robustness. The second stage is the pre-training of CNNs that is used for the deep feature extraction for obtaining the classification model.

The proposed model consists of an input image 32*32*1 initial convolution layer that learns the features of an image and the connections among the pixels would maintain. Mathematically, the deep learning classifier operates upon the filter and the matrix. Each convolution layer is supported by that of the ReLU sequence which is used for the mapping of output features. The ReLU function is given by

$$f\left(a_{0}\right)=max\left(0,a_{0}\right)$$

Then, in the next step, the image is fed into the pooling layer which reduces the dimensions of output feature maps, and also max pooling or average pooling is performed under this layer only. In the maximum pooling from the improved feature map, the largest component among them will be taken. The same way average pooling computes the average value of that area. Again, the pair of convolution layer along with ReLU gives the result of hidden 1024 layers. The dropout layer will lessen the training time results in malware families.

This method allows the deep learning classifier to work on the malware images using CNN; also, the CNN is used by the security professionals for training the models. The conversion of malware into an image is shown in Figure 7, where the input is in the form of binary then converted into 8-bit vector then converted into Grayscale, finally, the malware will be detected that is Alueron. gen!J malware.

There exists a variety of public malware datasets, one of them is the Malimg dataset that is being fed to CNN [72]. This particular dataset contains 25 malware families with 9339 samples.

After the feature selection steps, the extraction or use of any image characteristic for an example texture pattern, frequencies in image, intensity, or color features, with the use of several techniques such as Euclidean distance, or mean and standard deviation to generate the other feature vector is conducted. The obtained grayscale images after conversion from the malware feed the model into machine learning. To train the model, the grayscale images can be saved into NumPy, and also using different techniques such as SVM, k-means, and artificial neural networks the models can be trained. However, the most useful and the proposed classifier is the use of CNN. After completion of feature selection and engineering, CNN can be built. For our model, a convolutional network with two convolutional layers, with 32*32 inputs has been used. To build the model using Python libraries, with the previously installed TensorFlow and utils libraries it can be implemented.

### 5.4. Analysis and Discussion

This section discusses the various parameters along with the results achieved by applying the efficient lightweight CNN model. The performance metrics on which the dataset depends are as follows.

#### Performance Metrics

Performance metrics depend on how efficient the dataset used for achieving the best and secure results is. The first step followed is the training of the model by using the batch size which is 256, the cell size of 256, the dropout rate of 0.85 along the learning rate of 1e-3, the node size of [512, 256, 128] along with five layers. After the training of the model, the testing is performed as discussed in the above section. By doing this, a confusion matrix is obtained having Predicted Labels and True Labels. Based on these various parameters such as accuracy, recall (Rec), and precision (Prec) are obtained. The confusion matrix is shown in Table 5.

- **Accuracy:** “*The number of correct predictions over total number of True Positive predictions by the model is known as accuracy*”,
  where TP = True positive class prediction, TN = True Negative class prediction, FP = False positive class prediction, FN = False negative class prediction.
  (1) $$Accuracy=\frac{TP+T}{TP+TN+FP+F}.$$
- **Recall:** “*Number of true predictions over an actual number of true predictions made by the model is known as recall”*.
  (2) $$Recall=\frac{1}{C}\left(\sum_{c=1}^{C}\frac{TP_{c}}{TP_{c}+FN_{c}}\right).$$
- **Precision:** *“The actual true predictions over total true predictions made by the model known as precision”.*
(3) $$Precision=\frac{1}{C}\left(\sum_{c=1}^{C}\frac{TP_{c}}{TP_{c}+FP_{c}}\right).$$

**Figure 9:**
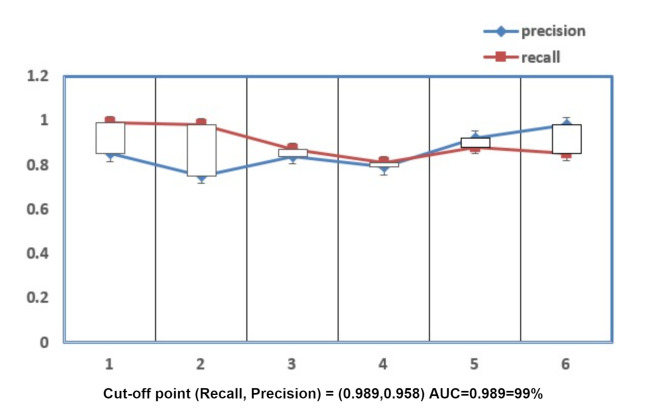
Recall and Precision.

**Figure 10:**
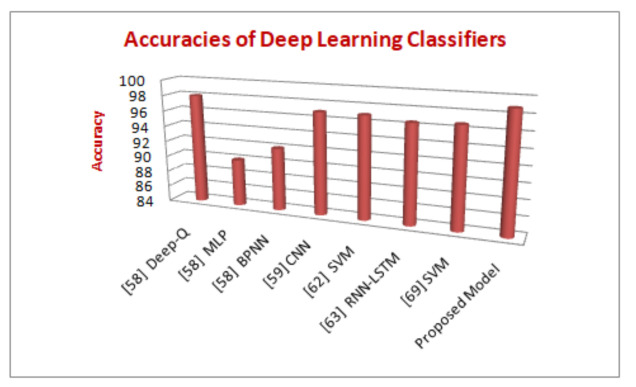
Comparison of state-of-the-art of deep learning models with the proposed model.

The results for Precision and Recall are described in Figure 9 as per the values discussed in confusion matrix in Table 4 which is between the number of samples and the malware families. The recall parameter shows the precise results in Adialer.c which is 0.989% The precision parameter is showing the best results in Agent.FYI, which is 0.958%. The values of precision, recall, and accuracy can be calculated by the formula mentioned above. These parameters are responsible for the evaluation of accuracy of the model. The accurate results shows how much secure is the proposed model. Therefore, as discussed above our proposed model is 99% secure in detection of Alueron.gen!J malware. The proposed model has shown the accuracy of 99% and is also compared with the other deep learning models, shown in Figure 10.

## 6. Conclusions

5G-IoT healthcare is an important field, and the security of sensitive information or personal records of the patient is of major importance. The deep neural network with CNN for maintaining security was proposed in this paper. The main focus of the parameters is on fully connected and pooling layers. To capture the different malware families of the image for the protection of sensitive data and information, an efficient method is used. The requirements for a model are to be fit for a fixed number of epochs 20, batch size of 64 samples, and exposure of 25 classes. The Keras deep learning library for deep learning convolutional neural networks is used for the implementation purpose. The model required a 32*32*1 input image for samples of 8 bit vectors, and grayscale vectors, which are loaded. Along with ReLU, the pooling layer and dropout layers are used to train and test the model. The proposed model has shown better accuracy over Deep-Q, MLP, BPNN, SVM, RNN-LSTM, DBN, etc. Achieving more accurate results for different types or combinations of deep learning techniques on various types of datasets is a further area of investigation.

## Figures and Tables

**Table 1:** 5G healthcare applications with its advantages and disadvantages.

5G Applications	Data-Bases	Keywords	Technique	Advantages	Disadvantages
TELE-MEDICINE SYSTEM [1]	NoSQL	IoT,Deep Learning	Qualitative Analysis	• No waiting time • Convenient • The patient is not able to catch the new disease • Non-dependency on others • No transportation required	• Lack in the face-to-face treatment • Protection of patient private data • A problem in case of emergency • Weak connectivity • Inability to examine the patient
ONLINE HEALTH MONITORING [3]	SQL and Wireless sensor networks,	Smart devices	Health Monitoring Architecture	• Good for post-operative apps. • Good Patient outcomes • Better quality of care	• Less Information exchange coordination • Connectivity should be good, not possible in rural areas. • The need for additional software
WTS [5]	Distri- buted Database	Security, Distributed	Master–Slave Architecture	• Suitable for the patients who cannot travel far • Less pain with quick recovery • Less trauma	• Expensive installation of the robotic surgery system • Latency • Failure in system components leads to the patient’s death
ONLINE CONSULTATION [19]	eXten- sible Mark-up Language	Doctor Consultation, Security and Privacy	Quanti- tative Approach	• Comfortable and convenient • Less infectious to patient • No waiting time	• Limited physical treatment as disease sometimes unclear • Network or communication issue
REMOTE DIAGNOSIS [29]	Distri- buted	Security, Smart Devices		• No stress being treated at home • Better utilization of ICU and wards • More patients attended by a specialized professional	• Lack of awareness and trust • Service centers are inadequate in number • Shortage of skilled healthcare doctors
AUGMEN- TED REALITY [31]		Privacy,IoT	Compre- hens ive Review	• Enhance user’s information • Real-time sharing over a long distance	• A little expensive • Lack of human interaction
VIRTUAL REALITY [33]	Central	Virtual reality	Cognitive Approach	• Can give results in an artificial environment • Detailed view • Effective Communication • Made education easy	• Cant deal with real-time as works virtually • Considering technology as an experiment • Expensive

**Table 2:** Security threats and its possible solutions.

Ref.No	Healthcare Application	Security Threats	Requirements	Possible Solutions
[7]	Online health monitoring	Denial-of-service	Availability	Redundancy Intrusion detection
[49]	Tele-medicine System	Unauthenticated and Unauthorized access	Key establishment	Public Key cryptography Random key distribution
[46]	Remote Diagnosis	Intrusion and high level attacks	ID	Secure group communication
[44]	M-health	Message manipulation	Authenticity and Integrity	Secure hash function and Digital signature
[51]	Online Consultation	Message disclosure	Privacy and Confidentiality	Access control Encryption
[39]	Augmented Reality	Compromised or attacked node	Resilient	Detection Tamper-proofing
[52]	Virtual Reality	Routing attacks	Secure routing	Secure protocols

**Table 3:** Comparison of accuracy of our proposed model with the state-of-the-art models.

Ref. No.	Deep Learning Classifier	Type of Attacks	Dataset	Accuracy
[59]	Deep Q Network	Malware Detection	DREBIN	98.79%
	Multi-layer perceptron			90.1%
	Back Propagation Neural Network			92.5%
[60]	Convolutional Neural Network	Spoofing,Sinkhole and Malicious Code	NSL-KDD	98.3%
[63]	Support Vector Machines	IoT authentication	KDD-CUP99	97.36%
	Multi-layer perceptron			98.40%
[64]	Recurrent Neural Network-Long Short-Term Memory	Malware Detection	DREBIN	98.18%
[51]	Deep Belief Network	Blackhole, DDoS, Wormhole	EMBER	98.47%
[69]	Support Vector Machines	DoS,	AAGM	96.7%
		False Data Injection		97.2%
		Replay attack		98.3%
		Man-in-the-middle		97.1%
Proposed Model	Convolutional Neural Network	Malware Detection	Malimg	99%

**Table 4:** Parameters and Values.

Parameter	Value
batch size	64
epoch	20
num classes	25

**Table 5:** Confusion matrix of Malimg.

Families	(1)	(2)	(3)	(4)	(5)	(6)	Prec	Rec
Adialer.C(1)	38	0	0	0	0	0	0.989	1.00
Agent.FYI(2)	0	44	0	0	0	0	1.00	0.956
Allaple.A(3)	0	0	852	0	0	0	0.84	0.87
Allaple.L (4)	0	0	1	477	0	0	0.79	0.81
Aleuron.gen!J(5)	0	0	0	0	68	0	0.92	0.88
Autorun.K(6)	0	0	0	0	0	68	0.998	0.978

## Data Availability

Not applicable.

## References

[1] Adebusola J.A., Ariyo A.A., Elisha O.A., Olubunmi A.M., Julius O.O. An Overview of 5G Technology. Proceedings of the ternational Conference in Mathematics, Computer Engineering and Computer Science (ICMCECS).

[2] Hossain M.S., Muhammad G. (2020). Deep Learning Based Pathology Detection for Smart Connected Healthcares. IEEE Netw..

[3] Latif S., Qadir J., Farooq S., Imran M.A. (2017). How 5g wireless (and concomitant technologies) will revolutionize healthcare?. Future Internet.

[4] Marescaux J., Leroy J., Rubino F., Smith M., Vix M., Simone M., Mutter D. (2002). Transcontinental robot-assisted remote telesurgery: Feasibility and potential applications. Ann. Surg..

[5] Tuli S., Wander G., Wander P., Gill S.S., Dustdar S., Sakellariou R., Rana O. (2020). Next generation technologies for smart healthcare: Challenges, vision, model, trends and future directions. Internet Technol. Lett..

[6] Shakeel P.M., Baskar S., Dhulipala V.S., Mishra S., Jaber M.M. (2018). Maintaining security and privacy in health care system using learning based deep-Q-networks. J. Med. Syst..

[7] Gubbi J., Buyya R., Marusic S., Palaniswami M. (2013). Internet of Things (IoT): A vision, architectural elements, and future directions. Future Gener. Comput. Syst..

[8] Swan M. (2015). Blockchain: Blueprint for a New Economy.

[9] Chen X.W., Lin X. (2014). Big data deep learning: Challenges and perspectives. IEEE Access.

[10] Tanwar S. (2020). Fog Computing for Healthcare 4.0 Environments.

[11] Akpakwu G.A., Silva B.J., Hancke G.P., Abu-Mahfouz A.M. (2017). A survey on 5G networks for the Internet of Things: Communication technologies and challenges. IEEE Access.

[12] Wang H., Fapojuwo A.O. (2017). A survey of enabling technologies of low power and long range machine-to-machine communications. IEEE Commun. Surv. Tutor..

[13] Rahman M.A., Hossain M.S., Loukas G., Hassanain E., Rahman S.S., Alhamid M.F., Guizani M. (2018). Blockchain-based mobile edge computing framework for secure therapy applications. IEEE Access.

[14] Hu L., Qiu M., Song J., Hossain M.S., Ghoneim A. (2015). Software defined healthcare networks. IEEE Wirel. Commun..

[15] Kumar B., Singh S.P., Mohan A. (2010). Emerging mobile communication technologies for health. Proceedings of the 2010 International Conference on Computer and Communication Technology (ICCCT).

[16] Hamdi O., Chalouf M.A., Ouattara D., Krief F. (2014). eHealth: Surveyon research projects, comparative study of telemonitoring architecturesand main issues. J. Netw. Comput. Appl..

[17] Alemdar H., Ersoy C. (2010). Wireless sensor networks for healthcare: A survey. Comput. Networks.

[18] Chen H., Abbas R., Cheng P., Shirvanimoghaddam M., Hardjawana W., Bao W., Li Y., Vucetic B. (2018). Ultra-reliable low latency cellular networks: Use cases, challenges and approaches. IEEE Commun. Mag..

[19] Lin D., Tang Y., Labeau F., Yao Y., Imran M., Vasilakos A.V. (2015). Internet of vehicles for e-health applications: A potential game for optimal network capacity. IEEE Syst. J..

[20] Soldani D., Fadini F., Rasanen H., Duran J., Niemela T., Chan-dramouli D., Nanavaty N. 5G Mobile Systems for Health-care. Proceedings of the 2017 IEEE 85th Vehicular Technology Conference (VTC Spring).

[21] De Mattos W.D., Gondim P.R. (2016). M-health solutions using 5G networks and M2M communications. IT Professional..

[22] Gupta R., Tanwar S., Tyagi S., Kumar N. (2019). Tactile-internet-based telesurgery system for healthcare 4.0: An architecture, research challenges, and future directions. IEEE Netw..

[23] Scarfone K., Souppaya M. (2013). Guidelines for Managing the Se-curity of Mobile Devices in the Enterprise. http://nvlpubs.nist.gov/nistpubs/SpecialPublications/NIST.SP.800–124r1.pdf.

[24] Mapp G., Aiash M., Ondiege B., Clarke M. Exploring a New SecurityFramework for Cloud Storage Using Capabilities. Proceedings of the 2014 IEEE 8th International Symposium on Service Oriented System Engineering.

[25] Vithanwattana N., Mapp G., George C. mHealth-Investigating an information security framework for mHealth data: Challenges and possible solutions. Proceedings of the 12th International Conference on Intelligent Environments (IE).

[26] Karmakar K.K., Varadharajan V., Tupakula U., Nepal S., Thapa C. Towards a Security Enhanced Virtualised Network Infrastructure for Internet of Medical Things (IoMT). Proceedings of the 2020 6th IEEE Conference on Network Softwarization (NetSoft).

[27] Hossain M.S., Muhammad G. (2017). Emotion-aware connected healthcare big data towards 5G. IEEE Internet Things J..

[28] Hossain M.S. (2015). Cloud-supported cyber–physical localization framework for patients monitoring. IEEE Internet Things J..

[29] Biswas S., Misra S. Designing of a prototype of e-health monitoring system. Proceedings of the 2015 In international Conference on Research in Computational Intelligence and Communication Networks.

[30] Brito J.M. Trends in wireless communications towards 5G networks—The influence of e-health and IoT applications. Proceedings of the 2016 International Multidisciplinary Conference on Computer and Energy Science (SpliTech).

[31] Sukhmani S., Sadeghi M., Erol-Kantarci M., El Saddik A. (2018). Edge caching and computing in 5G for mobile AR/VR and tactile internet. IEEE Multimed..

[32] Al Osman H., Eid M., El Saddik A. (2014). U-biofeedback: A multimedia-based reference model for ubiquitous biofeedback systems. Multimed. Tools Appl..

[33] Dananjayan S., Raj G.M. (2021). 5G in healthcare: How fast will be the transformation?. Ir. J. Med. Sci..

[34] Fang D., Qian Y., Hu R.Q. (2017). Security for 5G mobile wireless networks. IEEE Access.

[35] Azeez N.A., Van der Vyver C. (2019). Security and privacy issues in e-health cloud-based system: A comprehensive content analysis. Egypt Inform. J..

[36] Pussewalage H.S., Oleshchuk V.A. (2016). Privacy preserving mechanisms for enforcing security and privacy requirements in E-health solutions. Int. J. Inf. Manag..

[37] Gardašević G., Katzis K., Bajić D., Berbakov L. (2020). Emerging Wireless Sensor Networks and Internet of Things Technologies—Foundations of Smart Healthcare. Sensors.

[38] Zou Y., Zhu J., Wang X., Hanzo L. (2016). A survey on wireless security: Technical challenges, recent advances, and future trends. IEEE.

[39] Zhang Y., Zheng D., Deng R.H. (2018). Security and privacy in smart health: Efficient policy-hiding attribute-based access control. IEEE Internet Things J..

[40] Al-Janabi S., Al-Shourbaji I., Shojafar M., Shamshirband S. (2017). Survey of main challenges (security and privacy) in wireless body area networks for healthcare applications. Egypt Informatics J..

[41] Wang D., Chen D., Song B., Guizani N., Yu X., Du X. (2018). From IoT to 5G I-IoT: The next generation IoT-based intelligent algorithms and 5G technologies. IEEE Commun. Mag..

[42] Puppala M., He T., Yu X., Chen S., Ogunti R., Wong S.T. (2016). Data security and privacy management in healthcare applications and clinical data warehouse environment. Proceedings of the 2016 IEEE-EMBS International Conference on Biomedical and Health Informatics (BHI).

[43] Rahimi H., Zibaeenejad A., Safavi A.A. A novel IoT architecture based on 5G-IoT and next generation technologies. Proceedings of the 2018 IEEE 9th Annual Information Technology, Electronics and Mobile Communication Conference (IEMCON).

[44] Papaioannou M., Karageorgou M., Mantas G., Sucasas V., Essop I., Rodriguez J., Lymberopoulos D. (2020). A survey on security threats and countermeasures in internet of medical things (IoMT). Trans. Emerg. Telecommun. Technol..

[45] Sikder A.K., Babun L., Aksu H., Uluagac A.S. Aegis: A context-aware security framework for smart home systems. Proceedings of the 35th Annual Computer Security Applications Conference.

[46] Park Y., Son Y., Shin H., Kim D., Kim Y. This ain’t your dose: Sensor spoofing attack on medical infusion pump. Proceedings of the 10th USENIX Workshop on Offensive Technologies (WOOT 16).

[47] Liu J., Kwak K.S. Hybrid security mechanisms for wireless body area networks. Proceedings of the 2010 Second international Conference on Ubiquitous and Future Networks (ICUFN).

[48] Multiple Vulnerabilities in Animas Onetouch Pinginsulinpump. https://blog.rapid7.com/2016/10/04/r7–2016–07-multiplevulnerabilities-in-animas-onetouch-ping-insulin-pump/.

[49] Sundararajan T.V., Shanmugam A. (2010). A novel intrusion detection system for wireless body area network in health care monitoring. J. Comput. Sci..

[50] Algarni A. (2019). A survey and classification of security and privacy research in smart healthcare systems. IEEE Access.

[51] Thamilarasu G., Chawla S. (2019). Towards deep-learning-driven intrusion detection for the internet of things. Sensors.

[52] Otoum Y., Liu D., Nayak A. (2019). DL-IDS: A deep learning-based intrusion detection framework for securing IoT. Trans. Emerg. Telecommun. Technol..

[53] Patterson J., Gibson A. (2017). Deep Learning: A Practitioner’s Approach.

[54] Silver D., Huang A., Maddison C.J., Guez A., Sifre L., Van Den Driessche G., Schrittwieser J., Antonoglou I., Panneershelvam V., Lanctot M. (2016). Mastering the game of Go with deep neural networks and tree search. Nature.

[55] Krizhevsky A., Sutskever I., Hinton G.E. (2012). Imagenet classification with deep convolutional neural networks. Adv. Neural Inf. Process. Syst..

[56] Ma J., Sheridan R.P., Liaw A., Dahl G.E., Svetnik V. (2015). Deep neural nets as a method for quantitative structure–activity relationships. J. Chem. Inf. Model..

[57] Storcheus D., Rostamizadeh A., Kumar S. A survey of modern questions and challenges in feature extraction. Proceedings of the 1st International Workshop on Feature Extraction: Modern Questions and Challenges at NIPS 2015.

[58] Li H., Ota K., Dong M. (2018). Learning IoT in edge: Deep learning for the Internet of Things with edge computing. IEEE Netw..

[59] Yin X.C., Liu Z.G., Ndibanje B., Nkenyereye L., Riazul Islam S.M. (2019). An IoT-based anonymous function for security and privacy in healthcare sensor networks. Sensors.

[60] Newaz A.K., Sikder A.K., Rahman M.A., Uluagac A.S. (2020). A survey on security and privacy issues in modern healthcare systems: Attacks and defenses. arXiv.

[61] Islam S.R., Kwak D., Kabir M.H., Hossain M., Kwak K.S. (2015). The internet of things for health care: A comprehensive survey. IEEE Access.

[62] Lane N.D., Bhattacharya S., Georgiev P., Forlivesi C., Kawsar F. An early resource characterization of deep learning on wearables, smartphones and internet-of-things devices. Proceedings of the 2015 International Workshop on Internet of Things Towards Applications.

[63] Das R., Gadre A., Zhang S., Kumar S., Moura J.M. A deep learning approach to IoT authentication. Proceedings of the IEEE International Conference on Communications (ICC).

[64] HaddadPajouh H., Dehghantanha A., Khayami R., Choo K.K. (2018). A deep recurrent neural network based approach for internet of things malware threat hunting. Future Gener. Comput. Syst..

[65] Malasri K., Wang L. (2009). Design and implementation of a securewireless mote-based medical sensor network. Sensors.

[66] Rahman M.A., Hossain M.S., Islam M.S., Alrajeh N.A., Muhammad G. (2020). Secure and provenance enhanced internet of health things framework: A blockchain managed federated learning approach. IEEE Access.

[67] He D., Qiao Q., Gao Y., Zheng J., Chan S., Li J., Guizani N. (2019). Intrusion detection based on stacked autoencoder for connected healthcare systems. IEEE Netw..

[68] Zhang Q., Yang L.T., Chen Z., Li P. (2018). A survey on deep learning forbig data. Inf. Fusion.

[69] Newaz A.I., Sikder A.K., Babun L., Uluagac A.S. Heka: A novel intrusion detection system for attacks to personal medical devices. Proceedings of the 2020 IEEE Conference on Communications and Network Security (CNS).

[70] Habibzadeh H., Soyata T. (2020). Toward uniform smart healthcare ecosystems: A survey on prospects, security and privacy considerations. Connected Health in Smart Cities.

[71] Deng L., Abdel-Hamid O., Yu D. A deep convolutional neural network using heterogeneous pooling for trading acoustic invariance with phonetic confusion. Proceedings of the international Conference on Acoustics, Speech and Signal Processing.

[72] Kancherla K.S., Mukkamala S. Image visualization based malware detection. Proceedings of the 2013 IEEE Symposium on Computational Intelligence in Cyber Security (CICS).

